# p53 status and response to radiotherapy in rectal cancer: a prospective multilevel analysis

**DOI:** 10.1038/sj.bjc.6602622

**Published:** 2005-06-14

**Authors:** E Lopez-Crapez, F Bibeau, S Thézenas, M Ychou, J Simony-Lafontaine, A Thirion, D Azria, J Grenier, P Senesse

**Affiliations:** 1 Cancer Research Center, Val d'Aurelle Cancer Institute, Montpellier 34298, France; 2 Pathology Department, Val d'Aurelle Cancer Institute, Montpellier 34298, France; 3 Biostatistics Unit, Val d'Aurelle Cancer Institute, Montpellier 34298, France; 4 Medical and Digestive Oncology Department, Val d'Aurelle Cancer Institute, Montpellier 34298, France; 5 Radiotherapy Department, Val d'Aurelle Cancer Institute, Montpellier 34298, France

**Keywords:** p53 status, rectal cancer, radiotherapy, predictive marker

## Abstract

The aim of this study was to evaluate, in a prospective study, the predictive role of p53 status analysed at four different levels in identifying the response to preoperative radiotherapy in rectal adenocarcinoma. Before treatment, 70 patients were staged and endoscopic forceps biopsies from the tumour area were taken. p53 status was assessed by total cDNA sequencing, allelic loss analysis, immunohistochemistry, and p53 antibodies. Neoadjuvant treatment was based on preoperative radiotherapy or radiochemotherapy. Response to therapy was evaluated after surgery by both pathologic downstaging and histologic tumour regression grade. In all, 35 patients (50.0%) had p53 gene mutations; 44.4% of patients had an allelic loss; nuclear p53 overexpression was observed in 39 patients (55.7%); and p53 antibodies were detected in 11 patients (16.7%). In the multilevel analysis of p53 status, gene mutations correlated with both nuclear protein overexpression (*P*<0.0001) and loss of heterozygosity (*P*=0.013). In all, 29 patients (41.4%) were downstaged by pathologic analysis, and 19 patients (29.2%) were classified as tumour regression grade 1. Whatever the method of evaluation of treatment response, no correlation between p53 alterations and response to radiotherapy was observed. Our results do not support the use of p53 alterations alone as a predictive marker for response to radiotherapy in rectal carcinoma.

Preoperative radiotherapy for locally advanced rectal cancer is used to reduce local recurrence rates and to improve survival rates (Swedish Rectal Cancer Trial, 1997). The selection of patients for neoadjuvant therapy is currently based on clinical and pathological parameters, rectal endosonography, and CT-scan findings (Becouam *et al*, 1999). Nevertheless, these parameters do not predict the response to neoadjuvant therapy. Predictor markers of complete responses are thus needed for patients treated with preoperative radiotherapy.

The p53 tumour suppressor gene has been demonstrated to regulate cell cycle progression and apoptosis (Levine, 1997; Polyak *et al*, 1997). Particularly, p53 plays a major role in the cellular response to DNA damage and is an essential component of the pathway leading from DNA damage produced by ionising radiation to apoptosis (Canman *et al*, 1998; El Deiry, 2003). Several *in vitro* (Lowe *et al*, 1994; Bunz *et al*, 1999) and *in vivo* (Lee and Bernstein, 1993; Kemp *et al*, 2001) studies have demonstrated that p53 dysfunction might restrict therapeutic efficacy. However, depending on tumour site, type of therapy, and methods of p53 detection, controversial results have been obtained (Cote *et al*, 1997; Pai *et al*, 1998; Chiarugi *et al*, 1998; Pruschy *et al*, 2001).

p53 gene mutations have been found in a large number of human cancers (Hainaut *et al*, 1997) with a frequency of about 60% in rectal cancer (Hollstein *et al*, 1991). The status of p53 is frequently studied by immunohistochemistry (IHC). This method is rapid and applicable to large-scale samples, but the clinical value of this type of analysis is not always correlated with the data obtained by cDNA sequence analysis (Sjogren *et al*, 1996; Bazan *et al*, 2002). In addition, p53 autoantibodies (p53-Ab) have been found in the serum of patients with a variety of human neoplasms (Crawford *et al*, 1982; Soussi, 2000).

In this study, we attempted to assess whether a complete p53 status analysis, involving molecular, immunohistochemical, and serological studies, correlates with response to preoperative radiotherapy in a prospective study of 70 rectal carcinoma patients.

## MATERIALS AND METHODS

### Patients

In all, 70 patients with rectal adenocarcinoma were included in this study between 1996 and 2001. All of them were treated with preoperative radiotherapy or radiochemotherapy at the Val d’Aurelle Paul-Lamarque Cancer Institute in Montpellier. In this prospective study, the patients were staged using the 1997 TNM classification based on a clinical examination, endoscopic and endorectal ultrasonography evaluation, and computed tomography (CT) of the thorax and abdomen. There were 43 men and 27 women in this prospective study. The median age was 65 years (range 39–80 years). Clinicopathological characteristics of the 70 patients who underwent radiotherapy or radiochemotherapy are presented in Table 1. All patients had a life expectancy greater than 3 months and a WHO performance status of 0, 1, or 2. Informed consent was obtained from all patients before entering the study.

### Therapy

For radiotherapy, patients were treated in the supine position with a three-field isocentric technique using 18 MV photon beams daily, five times a week. The daily dose at the isocenter (in accordance with ICRU 62) was 1.8 Gy; the total dose to the entire pelvis was 45 Gy. In 29 patients, the primary tumour received a boost dose of up to 15 Gy, because a clinical response was observed during the conventional course of treatment. The pelvic target volume encompassed the posterior pelvis, the sacrum, the posterior half of the bladder, the prostate/vagina, and the presacral and low common iliac nodes up to the intervertebral space L4/L5. The boost volume covered the primary tumour plus a 2-cm margin using a three-field technique. Individually shaped blocks were used to shield normal tissues. Four patients received concurrent radiochemotherapy. The chemotherapy regimen consisted of continuous infusion of 5-fluorouracil (5-FU) and intravenous (i.v.) leucovorin beginning on the first day of radiation therapy. One patient received two cycles of 400 mg m^−2^ day^−1^ of 5-FU with 10 mg m^−2^ of leucovorin (days 1–5 and 29–33 of radiation therapy). Three patients received three 48-h courses of 5-FU (400 mg m^−2^ day^−1^) with 100 mg m^−2^ day^−1^ of leucovorin (days 1–2, 15–16, and 29–30 of radiation therapy). The median time between the first day of radiotherapy and surgery was 10 weeks (±3.9 weeks).

### Biopsy collection, processing, and histopathology

Endoscopic biopsies and rectal endosonography were performed by the same physician. All patients were given 250 ml of PEG enema before endorectal examination. Endorectal ultrasonography was performed with an endosonic linear probe (Model EUB-33, Hitachi). Patients were staged according to *u*TN criteria for ultrasound staging. Four pretherapeutic endoscopic biopsies from the macroscopic tumour area were performed in all cases. The endoscopic biopsies were frozen immediately after resection in liquid nitrogen (N_2_) and then embedded in Tissue-Tek®OCT compound (Sakura Finetech USA Inc., Torrance, CA, USA). Three consecutive 5-*μ*m-thick sections were cut. One slide was stained with haematoxylin and eosin to estimate the percentage of tumour cells, and the other slides were used for the IHC analyses. The remaining biological material was stored in liquid N_2_ until nucleic acid extraction. Two blood samples were collected from each patient on the day of clinical diagnosis for allelic loss and serological analyses.

### Nucleic acid extraction

DNA was extracted from the blood sample with QIAamp DNA Blood Maxi Kit (Qiagen, Hilden, Germany) according to the manufacturer's instructions. For biopsy samples, the OCT compound surrounding the tissue was discarded avoiding thawing in order to prevent RNA degradation. The biopsy was then placed in a polypropylene tube containing 800 *μ*l of extraction solution (TRIZOL® Reagent, Invitrogen, France) and homogenised using an Ultra-Turax apparutus. The RNA and DNA were then coextracted according to the manufacturer's recommendations. The RNA pellet was dissolved in 50 *μ*l of RNAse-free H_2_O, and 25 *μ*l were used for the cDNA synthesis. The DNA pellet was dissolved in 50 *μ*l of Tris-HCl buffer, pH 7.4.

### cDNA synthesis, PCR amplification, and direct sequencing

The tumour cDNA was obtained with a First-Strand cDNA Synthesis Kit (Amersham Pharmacia Biotech, Uppsala, Sweden) as specified by the manufacturer.

For all patients, the entire coding sequence (exons 2–11) was examined for p53 mutations by direct sequencing. Four sets of primers were designed to cover the entire p53 coding sequence. The four overlapping PCR fragments generated were then solid phase sequenced using an ALF express™ Automated DNA sequencer (Pharmacia Biotech, Sweden). PCR primers and sequencing oligonucleotides were synthesised based on the cDNA sequence of p53 as described previously (Thirion *et al*, 2002). Briefly, cDNA was subjected to PCR in a 50-*μ*l reaction mixture that contained 10 mmol l^−1^ Tris-HCl, pH 8.3, 50 mmol l^−1^ KCl, 0.1% Triton X-100, 1.5 mmol l^−1^ MgCl_2_, 1 U *Taq* DNA polymerase (Invitrogen, France), 6 pmol of each primer, and 200 *μ*mol l^−1^ of each dNTP. PCR were carried out in a Touch Down Thermocycler (Hybaid, UK) for 38 cycles: denaturation (15 s at 94°C), annealing (30 s at 58°C), and elongation (45 s at 72°C). A final 5-min elongation was performed at 72°C.

The Thermo Sequenase Fluorescent-Labelled Primer Cycle Sequencing Kit with 7-deaza-dGTP (Amersham Pharmacia Biotech) was used for direct sequencing as described previously (Thirion *et al*, 2002). Resolved sequencing products were analysed with the ALFwin Sequencer Analyser 2.00 software (Amersham Pharmacia Biotech); then, the SB mutation analyser software (Amersham Pharmacia Biotech) was used to compare the data obtained with the wild-type p53 sequence. Each mutation identified was confirmed by sequencing an entirely new PCR product, starting from the corresponding cDNA.

### Detection of loss of heterozygosity (LOH)

DNA was examined for LOH with four p53 intragenic markers: two restriction sites, namely, *Msp*I in inton 6 (McDaniel *et al*, 1991) and *Bst*UI in exon 4, (Ara *et al*, 1990), and two variable numbers of tandem repeats (Jones and Nakamura, 1992; Hahn *et al*, 1993) as described previously (Thirion *et al*, 2002). A patient was classified as informative when normal DNA demonstrated heterozygosity for one of the four markers. LOH was evaluated by comparing normal and tumour DNA band intensities by two independent readers. LOH was considered effective when the intensity of one allele in the tumour DNA represented less than 50% of the intensity of the other allele.

### IHC analysis

The overexpression of the p53 protein was evaluated on two consecutives 5-*μ*m frozen sections that were fixed in 50% methanol/acetone for 10 min at −20°C and then air-dried. Two monoclonal antibodies: Pab 1801 (1 : 25 dilution; Oncogene Research Products, Cambridge, MA, USA) and DO7 (1 : 25 dilution; Dako, Glostrup, Denmark) were used. The alkaline phosphatase and monoclonal anti-alkaline phosphatase staining procedure was used as described previously (Thirion *et al*, 2002). For each series of IHC analysis, a negative and a positive p53 control slide was included. Nuclear staining of the tumour tissues was scored as follows: <5, >5–10, >10–25, and >25%. A specimen was scored positive when more than 5% of the tumour cells showed nuclear staining with at least one of the anti-p53 antibodies (Pab 1801 or DO7). The same pathologist, blinded to the results of the other p53 analyses, reviewed all slides.

### Serological analysis

Serum p53-Ab were detected by a semiquantitative enzyme-linked immunosorbent assay commercially available (PharmaCell, France).

### Assessment of radiotherapy effects

Rectal endosonography is considered as the standard medical examination for parietal infiltration evaluation before treatment. Reliability determinations of this method, carried out by experts, is about 80 and 75% for *u*T and *u*N, respectively (Heriot *et al*, 1999). In our study, all staging were assessed by the same expert. Response to radiotherapy was based on both comparison of the *u*T stage *vs* the *p*T stage (pathologic downstaging) and histologic tumour regression grade of the surgical samples (Rectal Cancer Regression Grade (RCRG)). For the pathologic staging, patients were considered as responders to neoadjuvant therapy when a downstaging of one T stage was obtained. For the RCRG, the staging proposed by Wheeler *et al* (2002, 2004) was used. In the RCRG staging, RCRG 1=‘good’ responsiveness, with a sterilised tumour or the presence of remaining microscopic foci of adenocarcinoma; RCRG 2=marked fibrosis but with macroscopic tumour still present; and RCRG 3=‘poor’ response, little or no fibrosis in the presence of abundant macroscopic tumour. In our study, RCRG 1 patients were considered as histological responders, while histological non-responders corresponded to patients with RCRG 2 or RCRG 3. The same pathologist, blinded to the result of the p53 analysis, classified all tumours.

### Statistical analyses

To investigate the association between parameters, univariate statistical analyses were performed using Pearson's *χ*^2^ test with exact *P* computation for categorical variables or Fisher's exact test if applicable.

Multivariate analyses for response, by logistic regression, were carried out to evaluate the effect of interactions between the different variables.

Owing to the small number of patients and the fact that some patients did not have measurements for all variables, the power of analysis was reduced.

All *P*-values reported are two-sided. For all statistical tests, differences were considered as significant at the 5% level. Statistical analyses were performed on an IBM PC-compatible personal computer using the STATA 7.0 software.

## RESULTS

### Biological data on preradiotherapy samples

#### Pathological examinations of slides from pretreatment biopsies

In order to obtain a sufficient amount of tumour cells, a preliminary study was performed with three to six endoscopic biopsies from each rectal carcinoma patient. The quality of each endoscopic biopsy was evaluated by histopathological examination of a haematoxylin- and eosin-stained slide. For each patient, dysplasia lesions as well as specimens with <5% of tumour cells were excluded. We decided that four biopsies were necessary to carry out the complete p53 status analyses (sequencing, LOH, and IHC).

#### Total cDNA sequencing

Among the 70 patients analysed, 35 cases (50%) revealed p53 mutations (Table 2). All mutations but one were localised in the central region of p53, exons 5–8. There was a vast majority of point mutations (31 of 35, 88.6%) with 83.8% (26 of 31) of transitions and 16.2% of transversions (five of 31). In all, 16 (51.6%) of the 31 point mutations were located on the mutational hotspot codons 175 (31.2%), 245 (31.2%), 273 (18.7%), 248 (12.5%), and 282 (6.4%).

#### LOH analysis

Among 53 patients studied, the use of four intragenic markers allowed us to identify 45 informative cases (84.9%). LOH was detected in 20 patients (44.4%) and each LOH was confirmed by a new PCR.

#### Immunohistochemistry

All the 70 patients were analysed for p53 overexpression. A total of 31 patients (44.3%) were p53 IHC negative. p53 protein nuclear accumulation was detected in 39 cases with five patients (7.1%), three patients (4.2%) and 31 patients (44.4%) in categories >5−10, >10−25, and >25%, respectively.

#### Anti-p53 antibodies

Serum samples were obtained from 66 patients. In all, 11 patients (16.6%) were positive for the presence of p53-Ab. p53-Ab were recorded in 15.8% (three of 19), 14.9% (seven of 47), and 33.3% (one of three) of patients with *u*T2, *u*T3, and *u*T4 tumours, respectively. Among the 10 metastatic patients analysed for p53-Ab, four were positive.

#### Multilevel p53 analysis

Side-by-side univariate comparison of the four analyses: sequencing, LOH, IHC, and serology revealed a strong correlation between the sequencing results and immunohistochemical data (*P*<0.001) (Table 3). Nevertheless, seven patients were p53 mutated without p53 overexpression and 11 patients were positive immunohistochemically without any p53 mutation. A significant correlation between p53 LOH and p53 mutations was observed (*P*=0.013) (Table 3); mutated patients display majorly an LOH (65%), whereas 72% of nonmutated patients were LOH negative. Association between p53 antibodies positivity in the serum and p53 protein expression in the corresponding rectal carcinoma tissue was assessed. A trend to a correlation between the presence of p53 antibodies and p53 protein overexpression was observed: 72.7% of p53-Ab-positive patients had p53 tumour overexpression *vs* 56.3% of p53-Ab-negative patients. We noticed that one patient with circulating p53-Ab had no gene alteration and no p53 nuclear overexpression. One p53-Ab-positive patient was IHC positive with a wild-type gene, and one patient was IHC negative with a codon 175 mutation.

### Tumour response to preoperative radiotherapy

All patients underwent surgical resection after neoadjuvant treatment. Using the UICC TNM classification, a downstaging score was calculated for each patient by subtracting the ultrasonographic tumour stage (*u*T) from the pathologic tumour stage (*p*T). Downstaging scores were −3, −2, −1, 0, and 1 for one (1.4%), five (7.1%), 23 (32.9%), 37 (52.9%), and four patients (5.7%), respectively. In all, 29 patients (41.4%) were considered as responders (downstaging scores of −3, −2, and −1). In total, 41 patients (58.6%) were considered as non-responders (Table 4). Histopathological evaluation using RCRG staging could be performed on 65 patients. A total of 19 patients (29.2%) demonstrated a ‘good’ responsiveness. The *p*T stage correlated with the histological response (*P*=0.005) (Table 5). Nevertheless, among 12 patients staged as RCRG 1, five patients were staged *p*T2 and seven patients *p*T3. Moreover, a significant correlation was observed between pathologic downstaging and the RCRG stage (*P*=0.025).

#### Response to neoadjuvant therapy and clinicopathological parameters

We used a *χ*^2^ test to determine whether any of the clinicopathological covariates predicted the response to neoadjuvant therapy. By using pathologic downstaging, only gender and radiotherapy dose correlated with response (*P*<0.04 and *P*<0.05, respectively) (Table 6). In multivariate analysis, sex remained a significant predictor with an odds ratio of 0.33 (95% confidence interval (CI), 0.11–0.95; *P*=0.04). By using the histologic tumour regression grade, no correlation was observed.

#### Response to neoadjuvant therapy and p53 status

Uni- and multivariate analyses did not reveal any correlation between p53 abnormalities and response to therapy, whatever the staging used to evaluate tumour response (Tables 7 and 8).

Nevertheless, studies of the four p53 levels analysed on sterilised surgical samples (*p*T0N0) and microscopic residual tumours (*p*T1N0) *vs* other postsurgical stages demonstrated a trend to correlation (*P*=0.059) for LOH detection. Only 12% of patients (one of eight) with a *p*T0N0 or *p*T1N0 stage demonstrated an LOH, whereas for advanced stages, there was no difference.

## DISCUSSION

The aim of this prospective study was to assess the role of p53 complete analyses in the response to radiotherapy or radiochemotherapy in rectal carcinoma patients. Many other studies have searched for predictive criteria of tumour responsiveness to radiotherapy. In particular, the role of p53 has been investigated, but despite the clear contribution of p53 to the molecular pathogenesis of colorectal tumours, its role in the response to therapy is still unclear. Since the clinical ambiguity of p53 status is largely due to the method used to detect p53 abnormalities, a multilevel analysis of p53 (molecular, protein, and serological) was thus performed to detect all p53 alterations.

Endoscopic biopsies were used as starting material for sequencing, LOH determination, and protein overexpression studies. Owing to the small size of the biological specimens, an anatomopathological analysis of four different biopsies was performed to prevent contamination by normal cells, which would lead to sequencing or LOH misinterpretations. Biopsies with the highest cellularity and the highest number of tumour cells were selected.

In this study, 50% (35 of 70) of the patients demonstrated a p53 gene mutation. The vast majority of observed mutations (34 of 35) were found to be located in the p53 core domain, affecting the principal functions of p53 as transcription factor. These results are in agreement with other reports where p53 gene mutation rates in colorectal cancers varied from 30 to 63% (Iacopetta, 2003). This wide range of p53 gene alterations depends not only on the sensitivity of the technique used to detected mutations and the number of exons covered but also on the stage of development of the cancer lesions and the localisation of the tumour. Frequently, colorectal cancers are considered as a unique entity, but recent evidence suggests that different genetic pathways are involved in colorectal cancer (Frattini *et al*, 2004). Particularly, p53 mutation rates vary between the two forms of genomic instability associated with colorectal cancers: microsatellite instability and chromosomal instability (Tang *et al*, 2004).

Analysis of nuclear p53 protein accumulation was based on the use of two monoclonal antibodies, Pab 1801 and D07 (Baas *et al*, 1994). We detected 55.7% (39 of 70) of IHC-positive cases when the cutoff value for p53 positivity was set at 5%. The frequency of the positive p53 staining observed was consistent with reported data, ranging from 44.8 to 60.8% (Bosari *et al* 1994; Poller *et al*, 1997). In this study, association between p53 protein nucleic overexpression and p53 gene mutation was obtained for 28 patients (71.8%). This percentage is around 70% for colorectal cancer studies reported in the literature (Bosari *et al*, 1994). Our results revealed 25.7% of nonconcordant results with 11 IHC false-positives and seven IHC false-negatives. p53 protein overexpression analysed by IHC is the main technique used for the detection of p53 abnormalities in clinical specimens. Nevertheless, the equation ‘p53 DNA mutation=p53 protein overexpression’ has not always proven to be valid. Particularly, the immunohistochemical analysis had a 75% sensitivity and a 63% positive predictive value for p53 mutations (Greenblatt *et al*, 1994). IHC-false-negative cases could be due to nonsense mutations or genetic alterations unable to stabilise sufficiently the protein. On the other hand, IHC-false-positive samples may be attributed to (i) normal cell cycle fluctuations (Delmolino *et al*, 1993) when a low percentage of nuclear staining was observed or (ii) alternative stabilisation of the protein by alterations of p53 regulatory genes implicated directly in the negative feedback loop such as Mdm2 (Yin *et al*, 2002) or indirectly such as p14ARF (Esteller *et al*, 2000).

Overexpression of p53 protein in tumours induces an immune response, and among cancer patients, those with colorectal cancer have the highest prevalence of p53-Ab ranging from 13 to 32% (Angelopoulou *et al*, 1997). In our series, 16.6% of patients (11 of 66) had detectable levels of p53-Ab. The use of antibodies against p53 as serological marker in the clinical management of colorectal cancer patients has been reported, but the prognostic value of such antibodies (Kressner *et al*, 1998) and their potential use for prediction of curability (Takeda *et al*, 2001) or response to adjuvant chemotherapy (Lechpammer *et al*, 2004) are conflicting. Concerning the detection of p53-Ab in the serum of patients, in our study no clear correlation between tumour progression and p53-Ab presence was found. Lechpammer *et al* (2004) in a series of 220 colorectal cancer patients detected p53-Ab mainly in Dukes’ B and C stages. Moreover, Tang *et al* (2001), in a large study of 998 colorectal patients demonstrated that the presence of p53-Ab correlates with tumour progression in colorectal carcinogenesis and an increase with advanced node metastasis. However, in these studies, both colon and rectal cancers were analysed as a single entity, whereas our work was focalised only on rectal carcinoma.

Allelic loss of 17p is a frequent event associated with colorectal carcinogenesis (Baker *et al*, 1989). In most cancers, one allele carries a missense mutation and the other allele is lost (Baker *et al*, 1989; Nigro *et al*, 1989). Our series demonstrated that LOH at the p53 locus occurred in combination with sequence alterations. Nevertheless, disruption of p53 function has been described without any loss or inactivation of the intact allele (Inga *et al*, 1997; Gualberto *et al*, 1998).

As evaluation of response to therapy is still a matter of debate (Wheeler *et al*, 2002, 2004), we analysed response to therapy by both pathologic downstaging and histologic tumour regression grade. Our data demonstrated a significant correlation between downstaging and the RCRG stage. Two different doses of radiotherapy were used in our study population (45 and 60 Gy). Regarding p53 abnormalities, these two subgroups were similar. When downstaging was used, a significantly better response was observed for patients receiving high-dose irradiation. This correlation was lost when RCRG stage was used. This discordance may be related to patients demonstrating a ‘good’ responsiveness (RCRG 1), although residual tumour cells remained in the muscularis and mesorectal tissue leading to an absence of downstaging.

In our multilevel detection of p53 abnormalities, no p53 analysis showed a significant influence on response to preoperative irradiation when pretreatment tissues were analysed, whatever the method used to evaluate treatment response. In the literature, the role of p53 gene mutations in sensitivity or resistance to radiation therapy is still a subject of discussion (Chiarugi *et al*, 1998).

Rebischung *et al* (2002) showed by sequence analysis in a retrospective series of 86 rectal tumours with 41% responders that the presence of p53 mutations correlated with sensitivity to radiotherapy. However, Rodel *et al* (2002) analysed the histopathological response to radiotherapy in a series of 44 patients and demonstrated that neither the p53 nor the bcl-2 status was correlated with a response to radiotherapy, but they found that the apoptotic index may help to tailor therapy with regard to neoadjuvant treatment of rectal cancer. Similarly, Saw *et al* (2003), in a series of 60 low rectal tumours locally advanced, concluded that neither p53 by IHC and PCR–SSCP (single-strand conformation polymorphism), nor DCC (deleted in colon cancer) by IHC was associated with tumour downstaging.

Although no correlation was obtained for pretreated tissues in our study, a trend to correlation was observed on surgical samples where retention of heterozygosity was associated with *p*T0–*p*T1 stages.

When cells are exposed to ionising radiation, a complex response is initiated including cell cycle arrest in the G1 and the G2 phases, apoptosis, and DNA repair. Wild-type p53 is a cell cycle checkpoint determinant following irradiation (Kuerbitz *et al*, 1992); and in response to ionising radiation (Buschmann *et al*, 2000), p53 is stabilised through phosphorylation, inhibition of Mdm2-mediated degradation, and reduction in Mdm2 sumoylation. The consequence is promotion of either cell cycle arrest or apoptosis. Following gamma-irradiation-induced cell death, striking tissue specificity is observed, with distinct regulation of target p53-induced genes (Fei and El Deiry, 2003). Instead of static analyses, dynamic immunohistochemical studies, comparing expression of apoptosis-releated genes (Tannapfel *et al*, 1998; Rau *et al*, 2003) in pretherapy biopsies and the final resected specimen after neoadjuvant treatment, could contribute to molecular marker positioning. Furthermore, other components such as EGFR and cyclin D1 could play active roles in tumour response to radiotherapy (Milas *et al*, 2004).

Our analysis of rectal cancers investigated the implication of p53 dysregulation with relation to the response to neoadjuvant therapy. The strength of our study was three-fold. First, p53 was analysed at four different levels; second, it was a prospective study with the criteria for inclusion being (i) a pretreatment biopsy (ii), a complete course of radiotherapy, and (iii) surgical resection. Finally, two methods of response to treatment were used, namely, pathologic downstaging and histologic tumour regression grade. Our results based on a multilevel p53 analysis approach confirm that although p53 appears to be a major regulator, nevertheless it is certainly not the major indicator of tumour radiosensitivity.

## Figures and Tables

**Table 1:** Patient characteristics

**Parameter**	**Number of patients (%) (*n*=70)**
*Gender*	
Female	27 (38.6)
Male	43 (61.4)
	
*Median age*	65 years (39–80)
	
*Differentiation*	
Well	36 (51.4)
Moderately	28 (40.0)
Poorly	6 (8.6)
	
*uT*	
T1	1 (1.4)
T2	19 (27.1)
T3	47 (67.1)
T4	3 (4.3)
	
*uN*	
N−	39 (55.7)
N+	31 (44.3)
	
*Metastases*	
M−	59 (84.3)
M+	11 (15.7)
	
*Pretherapeutic TNM stage*	
Stage 1	11 (15.7)
Stage 2	23 (32.9)
Stage 3	25 (35.7)
Stage 4	11 (15.7)
	
*pT*	
0	4 (5.7)
1	6 (8.6)
2	26 (37.1)
3	34 (48.6)
	
*PN*	
0	48 (68.6)
1	20 (28.6)
2	2 (2.8)
	
*pTN stage*	
0	4 (5.7)
1	21 (30.0)
2	18 (25.7)
3	16 (22.5)
4	11 (15.7)
	
*Radiotherapy (Gy)*	
45	41 (59)
60	29 (41)

*u*T, *u*N=ultrasonographic tumour stage; *p*T, *p*N, *p*TN=pathologic tumour stage.

**Table 2:** Location and type of mutation in the p53 gene

**Number**	**Exon**	**Codon**	**Mutation**	**LOH**	**Serology**	**IHC**
810	7	248	CGG (Arg) → CAG (Gln)	−	−	+
100267	8	306	CGA (Arg) → TGA (Stop)	NA	NA	−
101644	8	273	CGT (Arg) → CAT (His)	−	+	+
101655	6	190	CCT → CC−del (3b)	NA	−	+
8200177	8	273	CGT (Arg) → TGT (Cys)	NI	−	+
9204069	5	175	CGC (Arg) → CAC (His)	NA	−	+
9601783	8	280	AGA → AG−del (6b)	NA	−	+
9602097	7	245	GGC (Gly) → GTC (Val)	+	+	+
9602836	5	164	AAG (Lys) → GAG (Glu)	NI	+	+
9603209	6	213	CGA (Arg) → TGG (Trp)	+	−	+
9605412	8	272	GTG (Val) → ATG (Met)	NI	−	+
9605686	8	273	CGT (Arg) → TGT (Cys)	+	−	+
9605908	8	272	GTG (Val) → TTG (Leu)	NI	−	+
9700728	7	242	TGC (Cys) → TAC (Tyr)	+	−	+
9700951	6	204	Ins (4b)	NA	−	−
9700984	7	245	GGC (Gly) → AGC (Ser)	+	−	+
9800986	5	175	CGC (Arg) → CAC (His)	−	+	−
9801082	8	284	CGG (Arg) → TGG (Trp)	+	−	−
9801303	5	175	CGC (Arg) → CAC (His)	+	−	+
9801337	6	213	CGA (Arg) → TGA (Stop)	NA	-	−
9801474	11	392	TCA → TC−del (4b)	NA	−	−
9803237	7	245	GGC (Gly) → AGC (Ser)	+	+	−
9803954	7	248	CGG (Arg) → CAG (Gln)	−	−	+
9804115	7	234	TAC (Tyr) → CAC (His)	+	−	+
9805071	5	175	CGC (Arg) → CAC (His)	+	−	+
9900522	7	245	GGC (Gly) → AGC (Ser)	NA	+	+
9902985	5	175	CGC (Arg) → CAC (His)	NA	−	+
9903004	8	281	CAG (Gln) → AAC (Asn)	NA	−	+
9903551	5	159	GCC (Ala) → GAC (Asp)	NI	−	+
9904431	5	158	CGC (Arg) → CAC (His)	+	−	+
9904676	5	161	GCC (Ala) → ACC (Thr)	+	+	+
9904929	8	282	CGG (Arg) → TGG (Trp)	+	−	+
9905447	5	163	TAC (Tyr) → AAC (Asn)	−	+	+
9905492	5	173	GTG (Val) → ATG (Met)	−	−	+
9905904	7	245	GGC (Gly) → AGC (Ser)	−	−	+

− LOH=no loss of heterozygosity; + LOH=loss of heterozygosity; − serology=absence of p53-Ab; + serology=presence of p53-Ab; − IHC=negative immunostaining; + IHC=positive immunostaining; NA=not available; NI=not informative.

**Table 3:** Univariate analysis of the four levels of p53 analysis

**Parameter**	**p53 mutated**	**p53 wild type**	***P* for trend**
*p53 IHC*			
Negative (*n*=31)	7 (23%)	24 (77%)	<0.001
Positive (*n*=39)	28 (72%)	11 (28%)	
			
*p53 LOH*			
No LOH (*n*=25)	7 (28%)	18 (72%)	0.013
LOH (*n*=20)	13 (65%)	7 (35%)	
			
*p53 serology*			
Negative (*n*=55)	26 (47%)	29 (53%)	NS
Positive (*n*=11)	8 (73%)	3 (27%)	

No LOH=retention of heterozygosity; NS=nonsignificant.

**Table 4:** Pathological response

	**Pathologic downstaging (%) (*n*=70)**			
**Echo *u*T**	***p*T0**	***p*T1**	***p*T2**	***p*T3**
*u*T1	0 (0%)	0 (0%)	1 (100%)	0 (0%)
*u*T2	3 (15.8%)	5 (26.3%)	8 (42.1%)	3 (15.8%)
*u*T3	1 (2.1%)	1 (2.1%)	16 (34%)	29 (61.7%)
*u*T4	0 (0%)	0 (0%)	1 (33.3%)	2 (66.7%)

**Table 5:** Pathologic downstaging compared with RCRG staging

	**RCRG (*n*=65)**	
**Pathologic staging**	**1**	**2+3**
*p*T0	3 (100%)	0 (0%)
*p*T1	4 (66.7%)	2 (33.3%)
*p*T2	5 (20.8%)	19 (79.2%)
*p*T3	7 (21.9%)	25 (78.1%)

RCRG=Rectal Cancer Regression Grade.

**Table 6:** Response to preoperative radiotherapy evaluated by downstaging according to the characteristics of the patients

**Parameter**	**Responders (*n*=29)**	**Non-responders (*n*=41)**	***P* for trend**
*Gender*			
Male	22	21	<0.04
Female	7	20	
			
*Age* (mean, s.d., years)	65.2±8.9	63.07±10.19	NS
			
*Differentiation*			
Well (*n*=36)	17 (47%)	19 (53%)	NS
Moderately (*n*=28)	11 (39%)	17 (61%)	
Poorly (*n*=6)	1 (17%)	5 (83%)	
			
*Pretherapeutic TNM stage*			
Stage 1 (*n*=11)	4 (36%)	7 (64%)	NS
Stage 2 (*n*=23)	10 (43%)	13 (57%)	
Stage 3 (*n*=25)	11 (44%)	14 (56%)	
Stage 4 (*n*=11)	4 (36%)	7 (64%)	
			
*Radiotherapy (Gy)*			
45 (*n*=41)	13 (32%)	28 (68%)	<0.05
60 (*n*=29)	16 (55%)	13 (45%)	

NS=nonsignificant.

**Table 7:** Response to preoperative radiotherapy evaluated by pathologic downstaging according to p53 alterations

**Variable**	**Responders**	**Nonresponders**	***P* for trend**
*p53 sequence*			
Wild type (*n*=35)	17 (49%)	18 (51%)	NS
Mutated (*n*=35)	12 (34%)	23 (66%)	
			
*p53 IHC*			
Negative (*n*=31)	9 (29%)	22 (71%)	NS
Positive (*n*=39)	20 (51%)	19 (49%)	
			
*p53 LOH*			
No LOH (*n*=25)	11 (44%)	14 (56%)	NS
LOH (*n*=20)	9 (45%)	11 (55%)	
			
*p53 serology*			
Negative (*n*=55)	22 (40%)	33 (60%)	NS
Positive (*n*=11)	7 (64%)	4 (36%)	

NS=nonsignificant; no LOH=retention of heterozygosity.

**Table 8:** Response to preoperative radiotherapy evaluated by RCRG according to p53 alterations

**Variable**	**RCRG 1**	**RCRG 2+RCRG3**	***P* for trend**
*p53 sequence*			
Wild type (*n*=31)	11 (35%)	20 (65%)	NS
Mutated (*n*=34)	8 (24%)	26 (76%)	
			
*p53 IHC*			
Negative (*n*=28)	8 (29%)	20 (71%)	NS
Positive (*n*=37)	11 (30%)	26 (70%)	
			
*p53 LOH*			
No LOH (*n*=23)	6 (26%)	17 (74%)	NS
LOH (*n*=19)	9 (47%)	10 (53%)	
			
*p53 serology*			
Negative (*n*=50)	17 (34%)	33 (66%)	NS
Positive (*n*=11)	2 (18%)	9 (82%)	

RCRG=Rectal Cancer Regression Grade; NS=nonsignificant; no LOH=retention of heterozygosity.

## References

[bib1] Angelopoulou K, Stratis M, Diamandis EP (1997) Humoral immune response against p53 protein in patients with colorectal carcinoma. Int J Cancer 70: 46–51 8985089 10.1002/(sici)1097-0215(19970106)70:1<46::aid-ijc7>3.0.co;2-6

[bib2] Ara S, Lee PS, Hansen MF, Saya H (1990) Codon 72 polymorphism of the TP53 gene. Nucleic Acids Res 18: 4961 10.1093/nar/18.16.4961 PMC332028 1975675

[bib3] Baas IO, Mulder JW, Offerhaus GJ, Vogelstein B, Hamilton SR (1994) An evaluation of six antibodies for immunohistochemistry of mutant p53 gene product in archival colorectal neoplasms. J Pathol 172: 5–12 7931827 10.1002/path.1711720104

[bib4] Baker SJ, Fearon ER, Nigro JM, Hamilton SR, Preisinger AC, Jessup JM, vanTuinen P, Ledbetter DH, Barker DF, Nakamura Y (1989) Chromosome 17 deletions and p53 gene mutations in colorectal carcinomas. Science 244: 217–221 2649981 10.1126/science.2649981

[bib5] Bazan V, Migliavacca M, Tubiolo C, Macaluso M, Zanna I, Corsale S, Amato A, Calo V, Dardanoni G, Morello V, La Farina M, Albanese I, Tomasino RM, Gebbia N, Russo A (2002) Have p53 gene mutations and protein expression a different biological significance in colorectal cancer? J Cell Physiol 191: 237–246 12064467 10.1002/jcp.10088

[bib6] Becouam Y, Blanc-Vincent MP, Lasser P, Dubois JB, Ducreux M, Giovannini M, Rougier P (1999) Standards, options and recommendations for the management of patients with primary adenocarcinoma of the rectum. National Federation of Centers for the Fight against Cancer. Presse Med 28: 1367–1374 10506869

[bib7] Bosari S, Viale G, Bossi P, Maggioni M, Coggi G, Murray JJ, Lee AK (1994) Cytoplasmic accumulation of p53 protein: an independent prognostic indicator in colorectal adenocarcinomas. J Natl Cancer Inst 86: 681–687 8158699 10.1093/jnci/86.9.681

[bib8] Bunz F, Hwang PM, Torrance C, Waldman T, Zhang Y, Dillehay L, Williams J, Lengauer C, Kinzler KW, Vogelstein B (1999) Disruption of p53 in human cancer cells alters the responses to therapeutic agents. J Clin Invest 104: 263–269 10430607 10.1172/JCI6863 PMC408422

[bib9] Buschmann T, Fuchs SY, Lee CG, Pan ZQ, Ronai Z (2000) SUMO-1 modification of Mdm2 prevents its self-ubiquitination and increases Mdm2 ability to ubiquitinate p53. Cell 101: 753–762 10892746 10.1016/s0092-8674(00)80887-9

[bib10] Canman CE, Lim DS, Cimprich KA, Taya Y, Tamai K, Sakaguchi K, Appella E, Kastan MB, Siliciano JD (1998) Activation of the ATM kinase by ionizing radiation and phosphorylation of p53. Science 281: 1677–1679 9733515 10.1126/science.281.5383.1677

[bib11] Chiarugi V, Magnelli L, Cinelli M (1998) Role of p53 mutations in the radiosensitivity status of tumor cells. Tumori 84: 517–520 9862508 10.1177/030089169808400501

[bib12] Cote RJ, Esrig D, Groshen S, Jones PA, Skinner DG (1997) p53 and treatment of bladder cancer. Nature 385: 123–125 10.1038/385123b0 8990112

[bib13] Crawford LV, Pim DC, Bulbrook RD (1982) Detection of antibodies against the cellular protein p53 in sera from patients with breast cancer. Int J Cancer 30: 403–408 6292117 10.1002/ijc.2910300404

[bib14] Delmolino L, Band H, Band V (1993) Expression and stability of p53 protein in normal human mammary epithelial cells. Carcinogenesis 14: 827–832 8504474 10.1093/carcin/14.5.827

[bib15] El Deiry WS (2003) The role of p53 in chemosensitivity and radiosensitivity. Oncogene 22: 7486–7495 14576853 10.1038/sj.onc.1206949

[bib16] Esteller M, Tortola S, Toyota M, Capella G, Peinado MA, Baylin SB, Herman JG (2000) Hypermethylation-associated inactivation of p14(ARF) is independent of p16(INK4a) methylation and p53 mutational status. Cancer Res 60: 129–133 10646864

[bib17] Fei P, El Deiry WS (2003) p53 and radiation responses. Oncogene 22: 5774–5783 12947385 10.1038/sj.onc.1206677

[bib18] Frattini M, Balestra D, Suardi S, Oggionni M, Alberici P, Radice P, Costa A, Daidone MG, Leo E, Pilotti S, Bertario L, Pierotti MA (2004) Different genetic features associated with colon and rectal carcinogenesis. Clin Cancer Res 10: 4015–4021 15217933 10.1158/1078-0432.CCR-04-0031

[bib19] Greenblatt MS, Bennett WP, Hollstein M, Harris CC (1994) Mutations in the p53 tumor suppressor gene: clues to cancer etiology and molecular pathogenesis. Cancer Res 54: 4855–4878 8069852

[bib20] Gualberto A, Aldape K, Kozakiewicz K, Tlsty TD (1998) An oncogenic form of p53 confers a dominant, gain-of-function phenotype that disrupts spindle checkpoint control. Proc Natl Acad Sci USA 95: 5166–5171 9560247 10.1073/pnas.95.9.5166 PMC20232

[bib21] Hahn M, Serth J, Fislage R, Wolfes H, Allhoff E, Jonas V, Pingoud A (1993) Polymerase chain reaction detection of a highly polymorphic VNTR segment in intron 1 of the human p53 gene. Clin Chem 39: 549–550 8448878

[bib22] Hainaut P, Soussi T, Shomer B, Hollstein M, Greenblatt M, Hovig E, Harris CC, Montesano R (1997) Database of p53 gene somatic mutations in human tumors and cell lines: updated compilation and future prospects. Nucleic Acids Res 25: 151–157 9016527 10.1093/nar/25.1.151 PMC146396

[bib23] Heriot AG, Grundy A, Kumar D (1999) Preoperative staging of rectal carcinoma. Br J Surg 86: 17–28 10027354 10.1046/j.1365-2168.1999.00996.x

[bib24] Hollstein M, Sidransky D, Vogelstein B, Harris CC (1991) p53 mutations in human cancers. Science 253: 49–53 1905840 10.1126/science.1905840

[bib25] Iacopetta B (2003) TP53 mutation in colorectal cancer. Hum Mutat 21: 271–276 12619112 10.1002/humu.10175

[bib26] Inga A, Cresta S, Monti P, Aprile A, Scott G, Abbondandolo A, Iggo R, Fronza G (1997) Simple identification of dominant p53 mutants by a yeast functional assay. Carcinogenesis 18: 2019–2021 9364015 10.1093/carcin/18.10.2019

[bib27] Jones MH, Nakamura Y (1992) Detection of loss of heterozygosity at the human TP53 locus using a dinucleotide repeat polymorphism. Genes Chromosomes Cancer 5: 89–90 1384667 10.1002/gcc.2870050113

[bib28] Kemp CJ, Sun S, Gurley KE (2001) p53 induction and apoptosis in response to radio- and chemotherapy *in vivo* is tumor-type-dependent. Cancer Res 61: 327–332 11196181

[bib29] Kressner U, Glimelius B, Bergstrom R, Pahlman L, Larsson A, Lindmark G (1998) Increased serum p53 antibody levels indicate poor prognosis in patients with colorectal cancer. Br J Cancer 77: 1848–1851 9667657 10.1038/bjc.1998.307 PMC2150331

[bib30] Kuerbitz SJ, Plunkett BS, Walsh WV, Kastan MB (1992) Wild-type p53 is a cell cycle checkpoint determinant following irradiation. Proc Natl Acad Sci USA 89: 7491–7495 1323840 10.1073/pnas.89.16.7491 PMC49736

[bib31] Lechpammer M, Lukac J, Lechpammer S, Kovacevic D, Loda M, Kusic Z (2004) Humoral immune response to p53 correlates with clinical course in colorectal cancer patients during adjuvant chemotherapy. Int J Colorectal Dis 19: 114–120 14634775 10.1007/s00384-003-0553-5

[bib32] Lee JM, Bernstein A (1993) p53 mutations increase resistance to ionizing radiation. Proc Natl Acad Sci USA 90: 5742–5746 8516323 10.1073/pnas.90.12.5742 PMC46798

[bib33] Levine AJ (1997) p53, the cellular gatekeeper for growth and division. Cell 88: 323–331 9039259 10.1016/s0092-8674(00)81871-1

[bib34] Lowe SW, Bodis S, McClatchey A, Remington L, Ruley HE, Fisher DE, Housman DE, Jacks T (1994) p53 status and the efficacy of cancer therapy *in vivo*. Science 266: 807–810 7973635 10.1126/science.7973635

[bib35] McDaniel T, Carbone D, Takahashi T, Chumakov P, Chang EH, Pirollo KF, Yin J, Huang Y, Meltzer SJ (1991) The MspI polymorphism in intron 6 of p53 (TP53) detected by digestion of PCR products. Nucleic Acids Res 19: 4796 10.1093/nar/19.17.4796-a PMC328758 1716362

[bib36] Milas L, Fan Z, Andratschke NH, Ang KK (2004) Epidermal growth factor receptor and tumor response to radiation: *in vivo* preclinical studies. Int J Radiat Oncol Biol Phys 58: 966–971 14967457 10.1016/j.ijrobp.2003.08.035

[bib37] Nigro JM, Baker SJ, Preisinger AC, Jessup JM, Hostetter R, Cleary K, Bigner SH, Davidson N, Baylin S, Devilee P (1989) Mutations in the p53 gene occur in diverse human tumour types. Nature 342: 705–708 2531845 10.1038/342705a0

[bib38] Pai HH, Rochon L, Clark B, Black M, Shenouda G (1998) Overexpression of p53 protein does not predict local-regional control or survival in patients with early-stage squamous cell carcinoma of the glottic larynx treated with radiotherapy. Int J Radiat Oncol Biol Phys 41: 37–42 9588915 10.1016/s0360-3016(98)00025-x

[bib39] Poller DN, Baxter KJ, Shepherd NA (1997) p53 and Rb1 protein expression: are they prognostically useful in colorectal cancer? Br J Cancer 75: 87–93 9000603 10.1038/bjc.1997.14 PMC2222690

[bib40] Polyak K, Xia Y, Zweier JL, Kinzler KW, Vogelstein B (1997) A model for p53-induced apoptosis. Nature 389: 300–305 9305847 10.1038/38525

[bib41] Pruschy M, Rocha S, Zaugg K, Tenzer A, Hess C, Fisher DE, Glanzmann C, Bodis S (2001) Key targets for the execution of radiation-induced tumor cell apoptosis: the role of p53 and caspases. Int J Radiat Oncol Biol Phys 49: 561–567 11173155 10.1016/s0360-3016(00)01480-2

[bib42] Rau B, Sturm I, Lage H, Berger S, Schneider U, Hauptmann S, Wust P, Riess H, Schlag PM, Dorken B, Daniel PT (2003) Dynamic expression profile of p21WAF1/CIP1 and Ki-67 predicts survival in rectal carcinoma treated with preoperative radiochemotherapy. J Clin Oncol 21: 3391–3401 12885834 10.1200/JCO.2003.07.077

[bib43] Rebischung C, Gerard JP, Gayet J, Thomas G, Hamelin R, Laurent-Puig P (2002) Prognostic value of p53 mutations in rectal carcinoma. Int J Cancer 100: 131–135 12115559 10.1002/ijc.10480

[bib44] Rodel C, Grabenbauer GG, Papadopoulos T, Bigalke M, Gunther K, Schick C, Peters A, Sauer R, Rodel F (2002) Apoptosis as a cellular predictor for histopathologic response to neoadjuvant radiochemotherapy in patients with rectal cancer. Int J Radiat Oncol Biol Phys 52: 294–303 11872273 10.1016/s0360-3016(01)02643-8

[bib45] Saw RP, Morgan M, Koorey D, Painter D, Findlay M, Stevens G, Clarke S, Chapuis P, Solomon MJ (2003) p53, deleted in colorectal cancer gene, and thymidylate synthase as predictors of histopathologic response and survival in low, locally advanced rectal cancer treated with preoperative adjuvant therapy. Dis Colon Rectum 46: 192–202 12576893 10.1007/s10350-004-6524-2

[bib46] Sjogren S, Inganas M, Norberg T, Lindgren A, Nordgren H, Holmberg L, Bergh J (1996) The p53 gene in breast cancer: prognostic value of complementary DNA sequencing versus immunohistochemistry. J Natl Cancer Inst 88: 173–182 8632491 10.1093/jnci/88.3-4.173

[bib47] Soussi T (2000) p53 Antibodies in the sera of patients with various types of cancer: a review. Cancer Res 60: 1777–1788 10766157

[bib48] Swedish Rectal Cancer Trial (1997) Improved survival with preoperative radiotherapy in resectable rectal cancer. N Engl J Med 336: 980–987 9091798 10.1056/NEJM199704033361402

[bib49] Takeda A, Shimada H, Nakajima K, Imaseki H, Suzuki T, Asano T, Ochiai T, Isono K (2001) Monitoring of p53 autoantibodies after resection of colorectal cancer: relationship to operative curability. Eur J Surg 167: 50–53 11213822 10.1080/110241501750069828

[bib50] Tang R, Changchien CR, Wu MC, Fan CW, Liu KW, Chen JS, Chien HT, Hsieh LL (2004) Colorectal cancer without high microsatellite instability and chromosomal instability – an alternative genetic pathway to human colorectal cancer. Carcinogenesis 25: 841–846 14729584 10.1093/carcin/bgh074

[bib51] Tang R, Ko MC, Wang JY, Changchien CR, Chen HH, Chen JS, Hsu KC, Chiang JM, Hsieh LL (2001) Humoral response to p53 in human colorectal tumors: a prospective study of 1,209 patients. Int J Cancer 94: 859–863 11745489 10.1002/ijc.1541

[bib52] Tannapfel A, Nusslein S, Fietkau R, Katalinic A, Kockerling F, Wittekind C (1998) Apoptosis, proliferation, bax, bcl-2 and p53 status prior to and after preoperative radiochemotherapy for locally advanced rectal cancer. Int J Radiat Oncol Biol Phys 41: 585–591 9635706 10.1016/s0360-3016(98)00076-5

[bib53] Thirion A, Rouanet P, Thezenas S, Detournay D, Grenier J, Lopez-Crapez E (2002) Interest of investigating p53 status in breast cancer by four different methods. Oncol Rep 9: 1167–1172 12375013

[bib54] Wheeler JM, Warren BF, Mortensen NJ, Ekanyaka N, Kulacoglu H, Jones AC, George BD, Kettlewell MG (2002) Quantification of histologic regression of rectal cancer after irradiation: a proposal for a modified staging system. Dis Colon Rectum 45: 1051–1056 12195189 10.1007/s10350-004-6359-x

[bib55] Wheeler JM, Dodds E, Warren BF, Cunningham C, George BD, Jones AC, Mortensen NJ (2004) Preoperative chemoradiotherapy and total mesorectal excision surgery for locally advanced rectal cancer: correlation with rectal cancer regression grade. Dis Colon Rectum 47: 2025–2031 15657650 10.1007/s10350-004-0713-x

[bib56] Yin Y, Stephen CW, Luciani MG, Fahraeus R (2002) P53 stability and activity is regulated by Mdm2-mediated induction of alternative p53 translation products. Nat Cell Biol 4: 462–467 12032546 10.1038/ncb801

